# Diffuse Unilateral Leg Swelling

**DOI:** 10.1016/j.acepjo.2025.100270

**Published:** 2025-10-25

**Authors:** Bruce M. Lo, Kylie Butler, Navjot Kaur, Kirsten Pennell

**Affiliations:** 1 Sentara Norfolk General Hospital/Eastern Virginia Medical School at Old Dominion University, Norfolk, Virginia, USA; 2 Eastern Virginia Medical School at Old Dominion University, Norfolk, Virginia, USA

**Keywords:** pyomyositis, leg swelling

## 1 Patient Presentation

**Figure 1:**
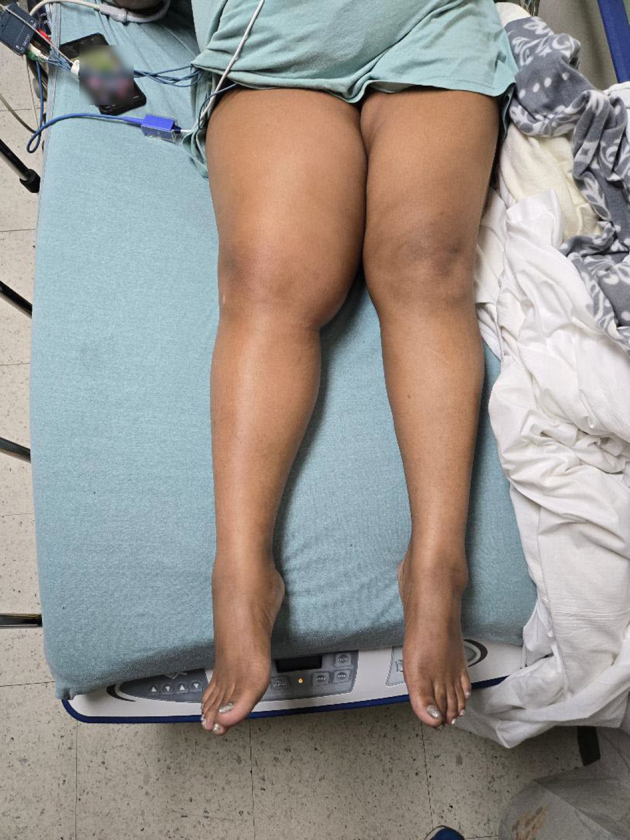
Diffuse right leg swelling with diffuse tenderness.

**Figure 2:**
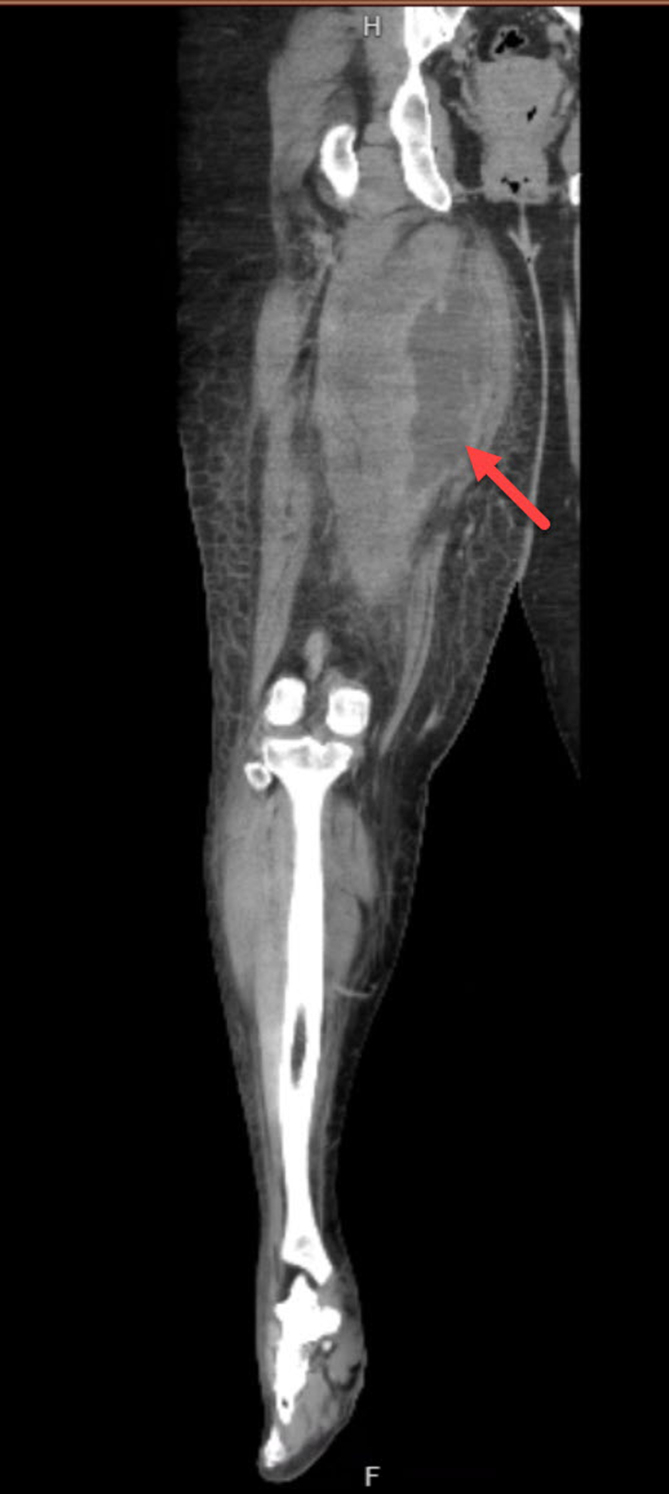
Computed tomography of right leg showing a 19 × 7 × 6.2 cm fluid collection (red arrow).

A 46-year-old woman with a history of hypertension presented with 14 days of right leg pain. The pain began after straining her leg while lifting heavy boxes at work, with diffuse leg swelling noted 3 days later. She reported subjective fever but no other complaints. Patient denies any illicit drug use. Initial vital signs included a temperature of 100.1 °F, heart rate of 122 beats per minute, and blood pressure of 158/91 mm Hg. Physical examination revealed a diffusely swollen and tender right leg (Fig 1). Computed tomography (CT) of the right leg identified a 19 × 7 × 6.2 cm fluid collection within the adductor brevis and magnus muscles (Fig 2).

## 2 Diagnosis: Spontaneous Pyomyositis

Pyomyositis is a rare bacterial infection of the skeletal muscle that typically results in the formation of an abscess. It is believed to involve hematogenous spread to the muscle following injury. Although traditionally considered a tropical disease, pyomyositis is now seen in nontropical regions and is associated with significant morbidity and mortality if left untreated.

Presentation can look similar to early skin infections such as cellulitis, abscess, or deep venous thrombosis and typically affect the larger muscles in the upper and lower extremities.^1^ Early diagnosis requires high clinical suspicion as there may be a lack of dermatologic findings to prompt imaging with either ultrasound or CT. Treatment ranges from percutaneous or surgical debridement with antimicrobial therapy. Open incision and drainage and interventional radiology drainage procedures are commonly utilized.

## Funding and Support

By *JACEP Open* policy, all authors are required to disclose any and all commercial, financial, and other relationships in any way related to the subject of this article as per ICMJE conflict of interest guidelines (see www.icmje.org). The authors have stated that no such relationships exist.

## Conflict of Interest

All authors have affirmed they have no conflicts of interest to declare.
